# Supplementary material for: Photoperiod controls vegetation phenology across Africa
Source: Commun Biol. 2019 Oct 25;2:391. doi: 10.1038/s42003-019-0636-7 (PMC6814729; doi:10.1038/s42003-019-0636-7)
Supplement: Supplementary file 1 — Supplementary Information [file 42003_2019_636_MOESM1_ESM.pdf]

Supplementary Information for

Photoperiod controls vegetation phenology across Africa

Supplementary information

Supplementary Table 1: Partial correlation coefficients between SOS and cumulative climatic drivers in the northern latitudes of Africa summed over a 90 day period in a step of 10 days (* means significant at *P*<0.05)

Northern Africa																
	Croplands				Grasslands				Shrublands				Woodlands			
Preseason	Rain	Solar	Temp	Photo	Rain	Solar	Temp	Photo	Rain	Solar	Temp	Photo	Rain	Solar	Temp	Photo
10	0.35*	0.12	-0.35*	0.71*	0.25*	-0.37*	-0.46*	0.51*	0.06	-0.00	-0.19*	0.97*	0.24*	0.29*	0.16*	0.77*
20	0.40*	0.08	-0.33*	0.79*	0.23*	-0.43*	-0.45*	0.53*	0.11	-0.08	-0.28*	0.96*	0.32*	0.31*	0.15*	0.69*
30	0.42*	-0.07	-0.34*	0.87*	0.34*	-0.50*	-0.38*	0.69*	0.20*	-0.11	-0.39*	0.95*	0.30*	0.31*	0.10*	0.66*
40	0.47*	-0.23*	-0.24*	0.93*	0.36*	-0.52*	-0.35*	0.78*	0.24*	-0.02	-0.39*	0.93*	0.24*	0.30*	0.09*	0.65*
50	0.48*	-0.29*	-0.19*	0.95*	0.36*	-0.53*	-0.36*	0.81*	0.28*	-0.01	-0.39*	0.92*	0.23*	0.28*	0.11*	0.64*
60	0.46*	-0.33*	-0.14*	0.96*	0.34*	-0.52*	-0.37*	0.85*	0.31*	-0.01	-0.40*	0.90*	0.10	0.22*	0.16*	0.66*
70	0.47*	-0.34*	-0.11*	0.97*	0.34*	-0.52*	-0.34*	0.88*	0.32*	-0.02	-0.42*	0.87*	0.06	0.24*	0.12*	0.66*
80	0.47*	-0.39*	-0.05	0.98*	0.34*	-0.51*	-0.33*	0.92*	0.33*	-0.10	-0.45*	0.84*	0.05	0.24*	0.12*	0.66*
90	0.46*	-0.40*	-0.05	0.98*	0.33*	-0.50*	-0.34*	0.93*	0.32*	-0.09	-0.45*	0.81*	-0.02	0.22*	0.14*	0.70*

Supplementary Table 2: Partial correlation coefficients between SOS and cumulative climatic drivers in the southern latitudes of Africa summed over a 90 day period in a step of 10 days (* means significant at *P*<0.05)

Southern Africa																
	Croplands (South-western)				Grasslands				Shrublands				Woodlands			
Preseason	Rain	Solar	Temp	Photo	Rain	Solar	Temp	Photo	Rain	Solar	Temp	Photo	Rain	Solar	Temp	Photo
10	0.13	0.10	-0.16*	-0.96*	0.07	0.02	-0.42*	0.98*	-0.13	0.27*	0.07	0.92*	-0.01	0.03	-0.06	0.95*
20	0.12	0.03	-0.15*	-0.96*	0.13	0.01	-0.34*	0.98*	-0.12	0.17*	0.12	0.94*	-0.04	0.19*	-0.19*	0.96*
30	0.11	0.04	-0.15*	-0.96*	0.16*	0.01	-0.27*	0.98*	-0.13	0.16*	0.11	0.94*	-0.09	0.28*	-0.13	0.96*
40	0.11	0.05	-0.16*	-0.96*	0.28*	0.04	0.01	0.98*	-0.14	0.19*	0.06	0.95*	-0.14	0.31*	-0.03	0.97*
50	0.08	0.04	-0.12	-0.96*	0.32*	0.13	0.09	0.98*	-0.11	0.24*	-0.02	0.96*	-0.14	0.30*	-0.05	0.93*
60	0.10	0.05	-0.10	-0.96*	0.34*	0.06	0.36*	0.98*	-0.14	0.29*	-0.07	0.97*	-0.11	0.28*	-0.19*	0.92*
70	0.03	0.05	-0.11	-0.96*	0.33*	0.03	0.15*	0.98*	-0.08	0.35*	-0.13	0.98*	-0.05	0.30*	-0.15*	0.91*
80	0.05	0.03	-0.11	-0.96*	0.32*	0.02	0.17*	0.98*	-0.04	0.37*	-0.24*	0.98*	-0.00	0.42*	-0.21*	0.90*
90	0.10	0.07	-0.08	-0.96*	0.33*	0.06	0.21*	0.98*	-0.09	0.38*	-0.26*	0.99*	-0.01	0.39*	-0.28*	0.86*

Supplementary Table 3: Partial correlation coefficients between SOS and cumulative climatic drivers in the extreme north of Africa summed over a 90 day period in a step of 10 days (* means significant at *P*<0.05)

Extreme North								
	Croplands				Shrublands			
Preseason	Rain	Solar	Temp	Photo	Rain	Solar	Temp	Photo
10	-0.08	-0.08	0.35*	-0.97*	0.26*	0.07	0.42*	-0.98*
20	-0.08	-0.09	0.34*	-0.98*	0.23*	-0.02	0.48*	-0.98*
30	-0.04	-0.10	0.31*	-0.98*	0.24*	-0.04	0.45*	-0.97*
40	-0.05	-0.08	0.23*	-0.98*	0.19*	-0.07	0.37*	-0.97*
50	-0.06	-0.12*	0.13*	-0.98*	0.20*	-0.06	0.21*	-0.97*
60	0.00	-0.15*	0.10*	-0.98*	0.22*	-0.02	0.08	-0.97*
70	0.05	-0.17*	0.07	-0.99*	0.25*	-0.04	0.00	-0.98*
80	0.10*	-0.20*	0.06	-0.99*	0.26*	-0.08	-0.03	-0.98*
90	0.15*	-0.22*	0.08	-0.99*	0.28*	-0.09	-0.07	-0.98*

Supplementary Table 4: Partial correlation coefficients between EOS DOY and cumulative climatic drivers in the northern latitudes of Africa summed over a 90 day period in a step of 10 days while controlling for SOS (* means significant at P<0.05).

Northern																				
	Croplands					Grasslands					Shrublands					Woodlands				
Presea	SOS	Rain	Solar	Temp	Photo	SOS	Rain	Solar	Temp	Photo	SOS	Rain	Solar	Temp	Photo	SOS	Rain	Solar	Temp	Photo
10	0.36*	-0.12*	0.36*	0.01	0.93*	0.08	-0.18*	-0.30*	0.32*	-0.86*	0.66*	-0.31*	0.08	0.00	-0.08	0.02	-0.33*	0.40*	0.29*	-0.07
20	0.37*	-0.19*	0.35*	0.02	0.92*	0.12*	-0.11*	-0.27*	0.45*	-0.86*	0.64*	-0.34*	0.07	-0.02	-0.18*	0.08	-0.44*	0.41*	0.09	-0.12*
30	0.39*	-0.22*	0.31*	0.04	0.88*	0.18*	-0.01	-0.31*	0.29*	-0.85*	0.62*	-0.34*	0.07	0.01	-0.27*	0.13	-0.51*	0.30*	0.02	-0.11*
40	0.42*	-0.24*	0.28*	0.04	0.83*	0.17*	0.09	-0.36*	0.26*	-0.87*	0.60*	-0.31*	0.09	-0.01	-0.36*	0.18*	-0.55*	0.18*	-0.10*	-0.13*
50	0.44*	-0.25*	0.27*	0.04	0.75*	0.18*	0.04	-0.33*	0.28*	-0.87*	0.57*	-0.29*	0.13	0.00	-0.45*	0.22*	-0.62*	0.09	-0.21*	-0.15*
60	0.47*	-0.26*	0.24*	0.02	0.65*	0.21*	-0.22*	-0.18*	0.15*	-0.84*	0.54*	-0.29*	0.16*	0.03	-0.52*	0.22*	-0.67*	0.01	-0.31*	-0.24*
70	0.47*	-0.29*	0.20*	0.00	0.51*	0.18*	-0.37*	-0.07*	0.13*	-0.83*	0.50*	-0.28*	0.21*	0.06	-0.58*	0.20*	-0.68*	-0.14*	-0.38*	-0.40*
80	0.44*	-0.36*	0.19*	0.00	0.42*	0.15*	-0.33*	-0.16*	-0.11*	-0.87*	0.46*	-0.35*	0.26*	0.10	-0.66*	0.22*	-0.67*	-0.14*	-0.39*	-0.50*
90	0.39*	-0.42*	0.16*	0.02	0.30*	0.16*	-0.29*	-0.14*	-0.19*	-0.87*	0.45*	-0.26*	0.28*	0.11	-0.70*	0.19*	-0.66*	-0.15*	-0.39*	-0.58*

Supplementary Table 5: Partial correlation coefficients between EOS DOY and cumulative climatic drivers in the southern latitudes of Africa summed over a 90 day period in a step of 10 days while controlling for SOS (* means significant at P<0.05).

Southern																				
	Croplands(South-western)					Grasslands					Shrublands					Woodlands				
Preseason	SOS	Rain	Solar	Temp	Photo	SOS	Rain	Solar	Temp	Photo	SOS	Rain	Solar	Temp	Photo	SOS	Rain	Solar	Temp	Photo
10	0.15*	-0.19	0.05	-0.26*	0.94*	-0.12	-0.12*	0.10*	0.46*	-0.29*	-0.14*	-0.16*	-0.39*	0.44*	-0.44*	0.19*	-0.44*	-0.02	0.35*	-0.46*
20	0.20*	-0.10	0.06	-0.30*	0.93*	-0.15	-0.11*	0.12*	0.57*	-0.18*	-0.17*	-0.10*	-0.40*	0.53*	-0.56*	0.12*	-0.36*	-0.04	0.33*	-0.50*
30	0.19*	-0.08	0.00	-0.21*	0.94*	-0.07	-0.21*	0.19*	0.57*	-0.15*	-0.21*	-0.16*	-0.36*	0.57*	-0.70*	0.19*	-0.28*	-0.03	0.32*	-0.52*
40	0.20*	-0.22	0.04	-0.20*	0.95*	0.00	-0.31*	0.30*	0.48*	-0.39*	-0.20*	-0.16*	-0.31*	0.57*	-0.75*	0.19*	-0.26*	-0.04	0.30*	-0.55*
50	0.20*	-0.12	0.05	-0.20*	0.95*	0.05	-0.33*	0.36*	0.38*	-0.59*	-0.18*	-0.15*	-0.28*	0.61*	-0.82*	0.16*	-0.33*	-0.04	0.30*	-0.58*
60	0.13*	-0.01	0.06	-0.13*	0.95*	0.09	-0.32*	0.34*	0.30*	-0.75*	-0.17*	-0.17*	-0.28*	0.65*	-0.87*	0.13*	-0.33*	-0.11	0.28*	-0.62*
70	0.10*	0.12	0.05	-0.13*	0.95*	0.12	-0.42*	0.34*	0.23*	-0.83*	-0.15*	-0.10*	-0.28*	0.70*	-0.91*	0.12*	-0.33*	-0.12	0.28*	-0.63*
80	0.11*	0.08	0.03	-0.18*	0.95*	0.07	-0.49*	0.30*	0.22*	-0.87*	-0.13*	0.04	-0.29*	0.76*	-0.94*	0.11*	-0.31*	-0.12	0.28*	-0.65*
90	0.08	0.13	-0.01	-0.24*	0.96*	0.02	-0.54*	0.25*	0.25*	-0.91*	-0.14*	0.08	-0.28*	0.77*	-0.95*	0.10*	-0.30*	-0.09	0.29*	-0.68*

Supplementary Table 6: Partial correlation coefficients between EOS and cumulative climatic drivers in the extreme north of Africa summed over a 90 day period in a step of 10 days, while controlling for SOS (* means significant at *P*<0.05).

Extreme North										
	Croplands					Shrublands				
Preseason	SOS	Rain	Solar	Temp	Photo	SOS	Rain	Solar	Temp	Photo
10	-0.03	-0.25*	-0.09	-0.75*	0.54*	0.05	-0.31*	-0.26*	-0.64*	0.29*
20	-0.06	-0.35*	0.01	-0.75*	0.65*	0.10*	-0.30*	-0.24*	-0.69*	0.05
30	-0.06	-0.39*	0.07	-0.72*	0.73*	0.20*	-0.34*	-0.27*	-0.75*	0.10
40	-0.05	-0.40*	0.12*	-0.67*	0.81*	0.21*	-0.38*	-0.08	-0.72*	0.26*
50	-0.04	-0.36*	0.18*	-0.65*	0.87*	0.19*	-0.43*	0.05	-0.77*	0.37*
60	-0.04	-0.32*	0.17*	-0.63*	0.92*	0.23*	-0.42*	0.18*	-0.76*	0.49*
70	-0.02	-0.28*	0.26*	-0.59*	0.94*	0.17*	-0.40*	0.29*	-0.72*	0.59*
80	-0.03	-0.29*	0.29*	-0.58*	0.95*	0.16*	-0.38*	0.32*	-0.68*	0.66*
90	-0.01	-0.21*	0.28*	-0.57*	0.96*	0.14*	-0.36*	0.37*	-0.63*	0.72*

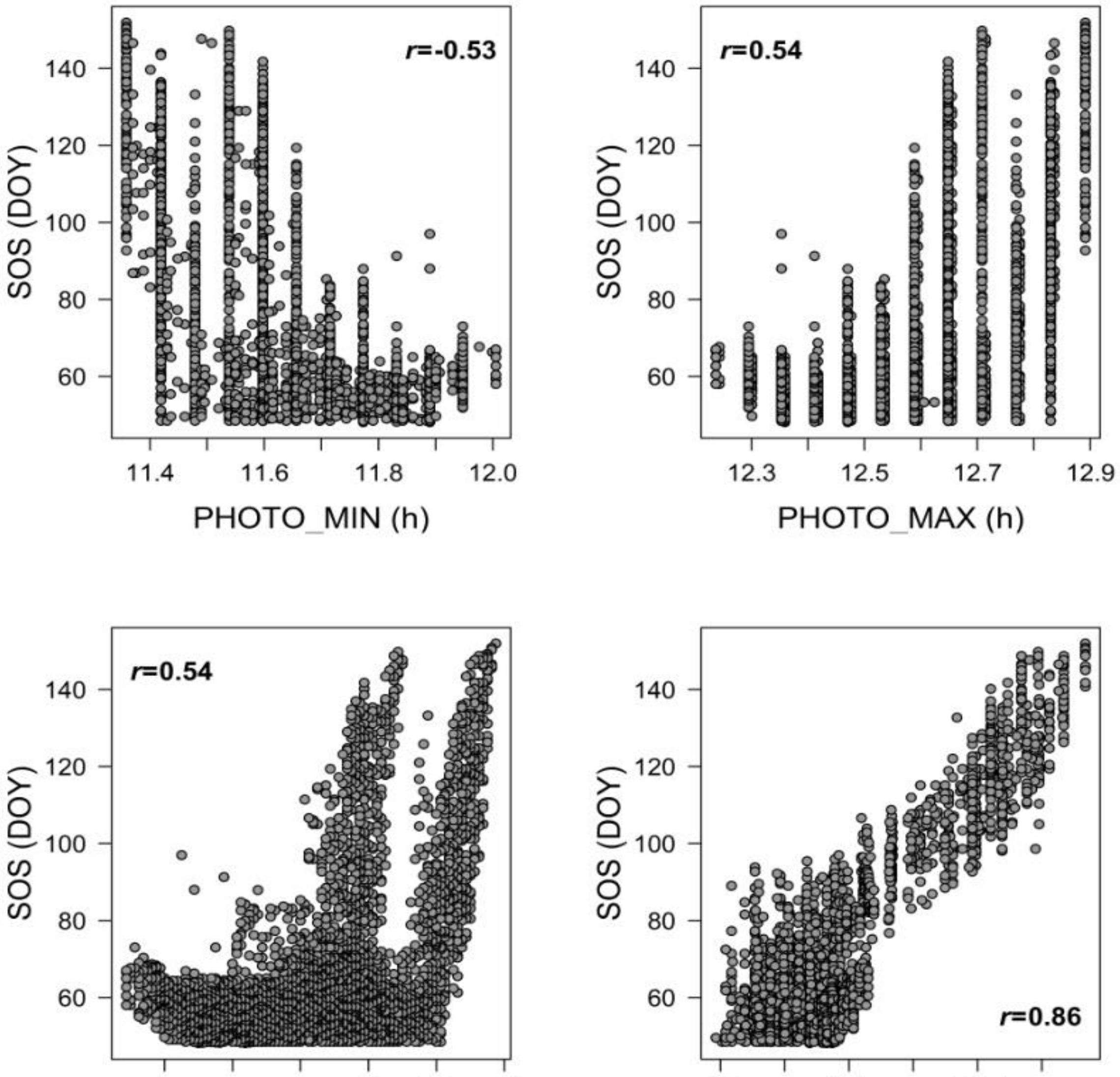

Supplementary Figure 1: Scatterplots between SOS and minimum and maximum photoperiod for the period 2001-to-2015, per degree increase in latitude, and 30 days preseason photoperiod across woodlands in the Northern hemisphere of Africa.

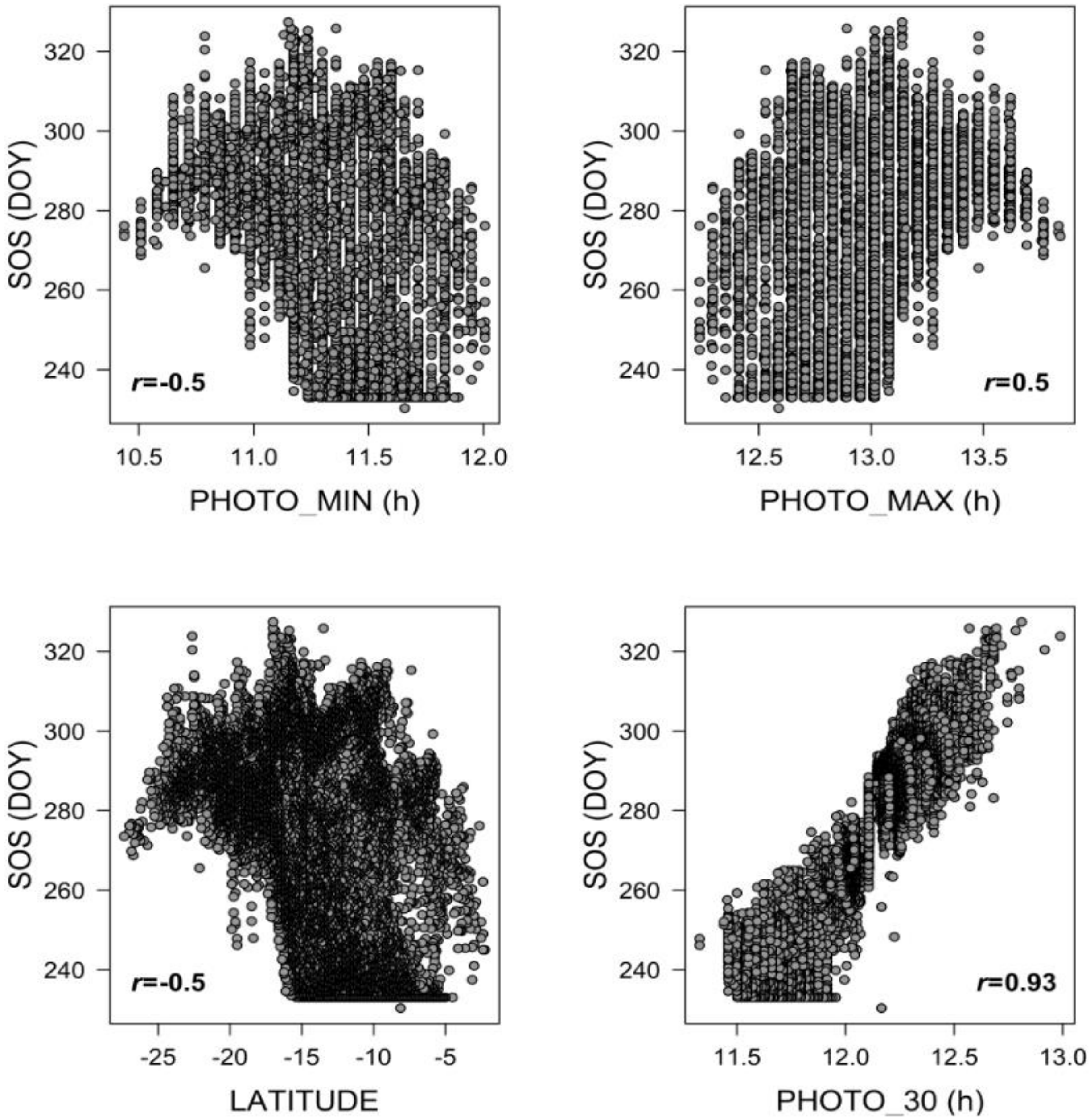

Supplementary Figure 2: Scatterplots between SOS and minimum and maximum photoperiod for the period 2001-to-2015, per degree increase in latitude, and 30 days preseason photoperiod across woodlands in the southern hemisphere of Africa.

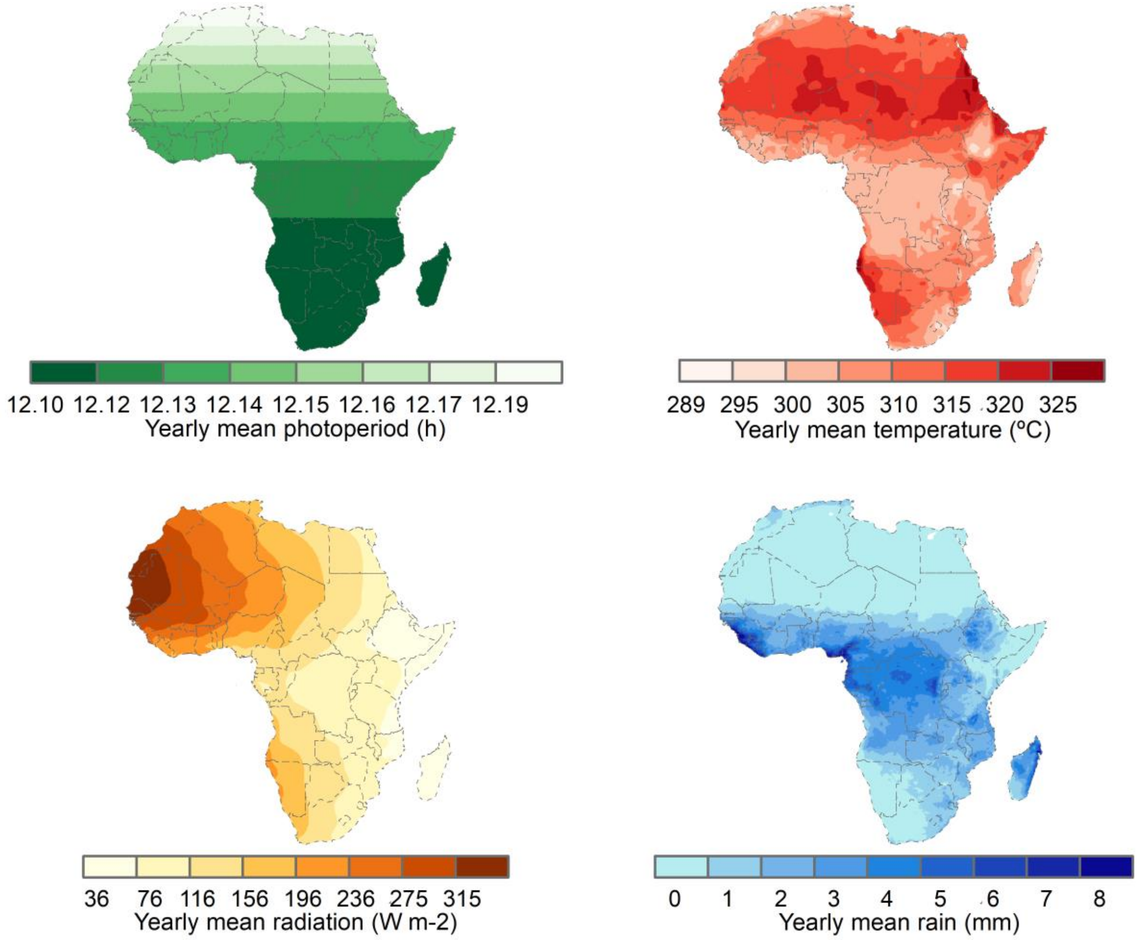

Supplementary Figure 3: Spatial distribution of 2016 yearly mean values of climatic factors.
