# Supplementary material for: Photoperiod controls vegetation phenology across Africa
Source: Commun Biol. 2019 Oct 25;2:391. doi: 10.1038/s42003-019-0636-7 (PMC6814729; doi:10.1038/s42003-019-0636-7)
Supplement: Supplementary file 2 — Reporting Summary [file 42003_2019_636_MOESM2_ESM.pdf]

nature research

Corresponding author(s): Tracy Adole, PhD

Last updated by author(s): May 16, 2019

Reporting Summary

Nature Research wishes to improve the reproducibility of the work that we publish. This form provides structure for consistency and transparency in reporting. For further information on Nature Research policies, see Authors & Referees and the Editorial Policy Checklist.

Statistics

For all statistical analyses, confirm that the following items are present in the figure legend, table legend, main text, or Methods section.

n/a	Confirmed	
☒	☐	The exact sample size (*n*) for each experimental group/condition, given as a discrete number and unit of measurement
☒	☐	A statement on whether measurements were taken from distinct samples or whether the same sample was measured repeatedly
☐	☒	The statistical test(s) used AND whether they are one- or two-sided<br>*Only common tests should be described solely by name; describe more complex techniques in the Methods section.*
☒	☐	A description of all covariates tested
☐	☒	A description of any assumptions or corrections, such as tests of normality and adjustment for multiple comparisons
☐	☒	A full description of the statistical parameters including central tendency (e.g. means) or other basic estimates (e.g. regression coefficient) AND variation (e.g. standard deviation) or associated estimates of uncertainty (e.g. confidence intervals)
☒	☐	For null hypothesis testing, the test statistic (e.g. *F*, *t*, *r*) with confidence intervals, effect sizes, degrees of freedom and *P* value noted<br>*Give P values as exact values whenever suitable.*
☒	☐	For Bayesian analysis, information on the choice of priors and Markov chain Monte Carlo settings
☒	☐	For hierarchical and complex designs, identification of the appropriate level for tests and full reporting of outcomes
☒	☐	Estimates of effect sizes (e.g. Cohen's *d*, Pearson's *r*), indicating how they were calculated

Our web collection on statistics for biologists contains articles on many of the points above.

Software and code

Policy information about availability of computer code

Data collection	Data was downloaded from the global online data archives of the LP DAAC (https://lpdaac.usgs.gov/); (http://chg.geog.ucsb.edu/data/chirps/) and (http://apps.ecmwf.int/datasets/) using the windows 10 command prompt and R 3.2.1 and R Studio version 1.0.153.
Data analysis	Data analysis was carried out with ESRI ArcMap and R 3.2.1 and R Studio version 1.0.153.

For manuscripts utilizing custom algorithms or software that are central to the research but not yet described in published literature, software must be made available to editors/reviewers. We strongly encourage code deposition in a community repository (e.g. GitHub). See the Nature Research guidelines for submitting code & software for further information.

Data

Policy information about availability of data

All manuscripts must include a data availability statement. This statement should provide the following information, where applicable:

- Accession codes, unique identifiers, or web links for publicly available datasets
- A list of figures that have associated raw data
- A description of any restrictions on data availability

The datasets generated during the current study are available in the University of Southampton Institutional Repository http://dx.doi.org/10.5258/SOTON/D0407

Field-specific reporting

Please select the one below that is the best fit for your research. If you are not sure, read the appropriate sections before making your selection.

☐ Life sciences ☐ Behavioural & social sciences ☒ Ecological, evolutionary & environmental sciences

Ecological, evolutionary & environmental sciences study design

All studies must disclose on these points even when the disclosure is negative.

Study description	This study using satellite data and methods carried out a systematic analysis of the relationship between vegetation phenological parameters and a range of climatic drivers, to provide increased understanding of the climate-driven vegetation phenology of Africa.
Research sample	Datasets used were:<br>1) Medium spatial resolution Moderate Resolution Imagining Spectrometer (MODIS):<br>a) 16 years Surface MODIS/Terra Reflectance 8-Day L3 Global 500 m data (MOD09A1)<br>b) 13 years and MODIS/Terra Land Cover Type Yearly L3 Global 500 m data (MCD12Q1)<br>2) 16 years daily 0.05O gridded rainfall Climate Hazards Group InfraRed Precipitation with Station (CHIRPS) dataset.<br>3) 16 years daily skin temperature and daily surface solar radiation downwards 0.125O European Centre for Medium-Range Weather Forecasts (ECMWF) ERA-Interim data.<br>4) 16 years daily photoperiod generated from using a standard equation based on latitude and day of year in the 'geosphere' package in R.
Sampling strategy	The number of years used was the sample size and this was determined based on data availability. The Moderate Resolution Imagining Spectrometer (MODIS) data and product only became available from 2002 =, the year one of it's satellite was launched.
Data collection	All datasets were downloaded via the Internet using the windows 10 command prompt and R 3.2.1 and R Studio version 1.0.153.
Timing and spatial scale	Datasets download were for the entire African continent and from 2000 – 2016.
Data exclusions	No dataset was excluded from the analysis
Reproducibility	All attempts to repeat data analysis were successful as this entails running computer codes with the already processed satellite data.
Randomization	This study did not involve any form of sample grouping, and it also did not carry out any statistical predictive modeling.
Blinding	This study did not collect data related to any of human participation as only satellite imagery was used for data analysis.

Did the study involve field work? ☐ Yes ☒ No

Reporting for specific materials, systems and methods

We require information from authors about some types of materials, experimental systems and methods used in many studies. Here, indicate whether each material, system or method listed is relevant to your study. If you are not sure if a list item applies to your research, read the appropriate section before selecting a response.

Materials & experimental systems

n/a	Involved in the study
☒	☐ Antibodies
☒	☐ Eukaryotic cell lines
☒	☐ Palaeontology
☒	☐ Animals and other organisms
☒	☐ Human research participants
☒	☐ Clinical data

Methods

n/a	Involved in the study
☒	☐ ChIP-seq
☒	☐ Flow cytometry
☒	☐ MRI-based neuroimaging
